# Familiality and SNP heritability of age at onset and episodicity in major depressive disorder

**DOI:** 10.1017/S0033291715000215

**Published:** 2015-02-20

**Authors:** P. Ferentinos, A. Koukounari, R. Power, M. Rivera, R. Uher, N. Craddock, M. J. Owen, A. Korszun, L. Jones, I. Jones, M. Gill, J. P. Rice, M. Ising, W. Maier, O. Mors, M. Rietschel, M. Preisig, E. B. Binder, K. J. Aitchison, J. Mendlewicz, D. Souery, J. Hauser, N. Henigsberg, G. Breen, I. W. Craig, A. E. Farmer, B. Müller-Myhsok, P. McGuffin, C. M. Lewis

**Affiliations:** 1 MRC Social Genetic and Developmental Psychiatry Centre, Institute of Psychiatry, Psychology and Neuroscience, King's College London, London, UK; 2 2nd Department of Psychiatry, Attikon General Hospital, University of Athens, Athens, Greece; 3 Department of Biostatistics, Institute of Psychiatry, Psychology and Neuroscience, King's College London, London, UK; 4 Centro de Investigación Biomédica en Red de Salud Mental CIBERSAM, University of Granada, Spain; 5 Dalhousie University Department of Psychiatry, Halifax, Nova Scotia, Canada; 6 MRC Centre for Neuropsychiatric Genetics and Genomics, Neuroscience and Mental Health Research Institute, Cardiff University, Cardiff, UK; 7 Barts and The London Medical School, Queen Mary University of London, London, UK; 8 Department of Psychiatry, University of Birmingham, Birmingham, UK; 9 Department of Psychiatry, Trinity Centre for Health Science, Dublin, Ireland; 10 Department of Psychiatry, Washington University, St. Louis, Missouri, USA; 11 Max Planck Institute of Psychiatry, Munich, Germany; 12 Department of Psychiatry, University of Bonn & German Center of Neurodegenerative Diseases (DZNE), Bonn, Germany; 13 Centre for Psychiatric Research, Aarhus University Hospital, Risskov, Denmark; 14 Division of Genetic Epidemiology in Psychiatry, Central Institute of Mental Health, Mannheim, Germany; 15 University Hospital Center and University of Lausanne, Lausanne, Switzerland; 16 Departments of Psychiatry and Medical Genetics, University of Alberta, Edmonton, Alberta, Canada; 17 Department of Psychiatry, Free University of Brussels, Brussels, Belgium; 18 Centre Européen de Psychologie Médicale PSY-PLURIEL, Bruxelles, Belgium; 19 Department of Genetics in Psychiatry, Poznan University of Medical Sciences, Poznan, Poland; 20 Department of Psychiatry, University of Zagreb, Zagreb, Croatia; 21 NIHR Biomedical Research Centre for Mental Health, South London and Maudsley NHS Foundation Trust and Institute of Psychiatry, Psychology and Neuroscience, King's College London, London, UK; 22 Division of Genetics and Molecular Medicine, King's College London, London, UK

**Keywords:** Age at onset, episodicity, familiality, GCTA, heritability, major depression

## Abstract

**Background:**

Strategies to dissect phenotypic and genetic heterogeneity of major depressive disorder (MDD) have mainly relied on subphenotypes, such as age at onset (AAO) and recurrence/episodicity. Yet, evidence on whether these subphenotypes are familial or heritable is scarce. The aims of this study are to investigate the familiality of AAO and episode frequency in MDD and to assess the proportion of their variance explained by common single nucleotide polymorphisms (SNP heritability).

**Method:**

For investigating familiality, we used 691 families with 2–5 full siblings with recurrent MDD from the DeNt study. We fitted (square root) AAO and episode count in a linear and a negative binomial mixed model, respectively, with family as random effect and adjusting for sex, age and center. The strength of familiality was assessed with intraclass correlation coefficients (ICC). For estimating SNP heritabilities, we used 3468 unrelated MDD cases from the RADIANT and GSK Munich studies. After similarly adjusting for covariates, derived residuals were used with the GREML method in GCTA (genome-wide complex trait analysis) software.

**Results:**

Significant familial clustering was found for both AAO (ICC = 0.28) and episodicity (ICC = 0.07). We calculated from respective ICC estimates the maximal additive heritability of AAO (0.56) and episodicity (0.15). SNP heritability of AAO was 0.17 (*p* = 0.04); analysis was underpowered for calculating SNP heritability of episodicity.

**Conclusions:**

AAO and episodicity aggregate in families to a moderate and small degree, respectively. AAO is under stronger additive genetic control than episodicity. Larger samples are needed to calculate the SNP heritability of episodicity. The described statistical framework could be useful in future analyses.

## Introduction

Despite extensive research in the field, the genetic architecture of major depressive disorder (MDD) remains highly elusive. Eight genome-wide association studies (GWAS) of MDD have been published (Sullivan *et al.* 2009; Lewis *et al.* 2010; Muglia *et al.* 2010; Rietschel *et al.* 2010; Kohli *et al.* 2011; Shi *et al.* 2011; Shyn *et al.* 2011; Wray *et al.* 2012), with only one locus of genome-wide significance (Kohli *et al.* 2011). A recently published mega-analysis of GWAS studies in MDD by the Psychiatric Genomics Consortium (PGC) failed to identify any genome-wide significant findings (PGC, 2013). Phenotypic and genetic heterogeneity have been pinpointed as partly responsible for these as yet unfruitful investigations.

Promising strategies to dissect MDD heterogeneity have mainly relied on subphenotypes such as age at onset (AAO) and recurrence/episodicity. Many researchers have used samples enriched in recurrent and early-onset forms (Shi *et al.* 2011; PGC, 2013) as these clinical subtypes are most consistently associated with higher familial aggregation and heritability of MDD (Sullivan *et al.* 2000). Other studies have directly focused on genetic correlates of the specific subphenotypes (AAO, episode frequency), analyzed as continuous traits (Power *et al.* 2012; Ferentinos *et al.* 2014). Yet, evidence on whether these subphenotypes are *per se* familial or heritable is scarce in MDD. Small or nil heritabilities for AAO and episode count, respectively, were reported in a small sample of 176 female twin pairs with MDD (Kendler *et al.* 1992).

Classical methods to estimate the narrow-sense heritability of quantitative traits, i.e. the percentage of phenotypic variance explained by additive genetic effects (*V*_A_/*V*_P_), rely on observed familial aggregation or phenotypic resemblance among relatives (parents–offspring, full-siblings, half-siblings, twins), usually on the basis of non-verifiable assumptions (random mating, absence of significant dominance, epistatic, and shared environmental effects or gene–environment correlation and interaction) (Visscher *et al.* 2008; Tenesa & Haley, 2013). The recently developed genome-wide complex trait analysis (GCTA) software (Yang *et al.* 2011) employs observed genetic covariance calculated from GWAS data in restricted maximum-likelihood linear mixed models (GREML) for unrelated subjects to estimate the percentage of phenotypic variance explained by common single nucleotide polymprphisms (i.e. the SNP heritability of a trait), without having to rely on those assumptions; SNP heritability estimates provide a lower bound on the total narrow-sense heritability of a phenotype (Zaitlen & Kraft, 2012).

Since subtyping is increasingly used in MDD genetic research, more evidence on the familial aggregation and heritability of subphenotypes, such as AAO and episodicity (which together reflect the disorder's temporal profile) would be valuable. The aims of this study are, therefore: first, to investigate the familiality of AAO and episode frequency of MDD (studied as quantitative/continuous phenotypes) and thereby infer estimates of their narrow-sense heritability (i.e. the proportion of the variance of AAO and episode frequency in MDD cases explained by additive genetic effects); second, to assess the SNP heritability of these subphenotypes in unrelated subjects with MDD.

## Subjects and method

### Samples

For the investigation of familial effects, we used subjects from the DeNt (Depression Network) affected siblings study (Farmer *et al.* 2004; McGuffin *et al.* 2005; Breen *et al.* 2011), which comprises cases of recurrent depression fulfilling DSM-IV and/or ICD-10 criteria of at least moderate severity ascertained from three UK clinical sites (London, Cardiff, Birmingham), four other European sites (Aarhus, Bonn, Dublin, Lausanne) and a site in St Louis, USA. One familial cluster of affected full siblings was identified in each family; in extended families, we used only the sibship including the proband or the sibship with most complete data on AAO and episode frequency. A total of 1498 subjects from 691 families with 2–5 affected full siblings were extracted for statistical analysis (Table 1).

**Table 1.** Composition of the DeNt affected full-siblings sample

Number of affected siblings per family	Number of families	Number of affected individuals (%)
2	600	1200 (80.1)
3	73	219 (14.6)
4	11	44 (2.9)
5	7	35 (2.3)
Total	691	1498 (100.0)

For the assessment of the SNP heritability of AAO, we used 3468 genotyped, unrelated MDD cases with complete data on age and AAO; 2695 cases were obtained from the RADIANT study, i.e. from the Depression Case-Control (DeCC) (*N* = 1023), DeNt (*N* = 843), GSK Case-Control (*N* = 140) and GENDEP (*N* = 689) studies, while 773 cases were obtained from the GSK Munich study. The DeCC study includes cases of recurrent depression of at least moderate severity ascertained from three UK sites (London, Cardiff, Birmingham) (Cohen-Woods *et al.* 2009). One proband from each DeNt family was genotyped and also included in the RADIANT study. The GSK Case-Control study includes cases of recurrent depression collected in Bonn and Lausanne in collaboration with GSK, using exactly the same protocol as the DeNt study. Cases from GENDEP (Uher *et al.* 2009), a pharmacogenetic study, were ascertained from various sites across Europe (London, Brussels, Mannheim, Bonn, Brescia, Aarhus, Ljubljana, Poznan, Zagreb); while recurrence was not a requirement, 59.5% of GENDEP cases suffered from recurrent MDD. The GSK Munich sample includes cases of recurrent depression of at least moderate severity recruited for a case-control study in the Munich area in collaboration with GSK (Tozzi *et al.* 2008; Muglia *et al.* 2010). To calculate the SNP heritability of episodicity, we extracted a subset of 2368 cases with recurrent MDD from the total sample with complete data on episode count (as well as on age and AAO), i.e. 994 DeCC, 833 DeNt, 139 GSK Case-Control and 402 GSK Munich cases.

In the RADIANT studies, only adults of European ancestry were recruited. Subjects were excluded if there was a history or family history (in first- or second-degree relatives) of schizophrenia, schizoaffective disorder or bipolar disorder, if they had experienced mood-incongruent psychotic symptoms, or if mood symptoms were solely related to alcohol or substance misuse or only secondary to medical illness or medication. Inclusion/exclusion criteria in the GSK Munich study were identical to those used in the DeCC and DeNT studies, except that subjects with a family history of bipolar disorder were not excluded. Therefore, we removed the latter from the analyses.

All subjects were interviewed with the Schedules for Clinical Assessment in Neuropsychiatry (SCAN; Wing *et al.* 1990), focusing on their worst and second-worst episodes of depression. AAO was recorded in SCAN items 1.016, 1.046–1.048 and 6.025; episode count was recorded in SCAN items 1.053 and 6.030. Family history of MDD in first-degree relatives was also extracted from SCAN item 1.045. All study participants provided written informed consent and approval was obtained from local ethics committees.

Whole-genome genotyping in the RADIANT sample was performed using the Illumina HumanHap610-Quad BeadChip. Genotyping in the GSK Munich sample was performed on the Illumina Human-Hap550K platform. Stringent quality control procedures were applied to individual and SNP data in both studies leaving a total of 471 581 and 511 503 SNPs, respectively, finally eligible for analysis (for full details see Lewis *et al.* 2010; Muglia *et al.* 2010). The two samples were then merged using PLINK v. 1.07 (Purcell *et al.* 2007) and taking into account flipped strands issues; the merged sample, including 427 946 SNPs shared by the original samples, was finally used with GCTA software.

## Ethical standards

The authors assert that all procedures contributing to this work comply with the ethical standards of the relevant national and institutional committees on human experimentation and with the Helsinki Declaration of 1975, as revised in 2008.

## Method

### Statistical modeling

Descriptive statistics were used to investigate the distributions of all variables in the DeNt families and genotyped case sets. AAO was analyzed as a continuous variable in linear mixed models; however, as it had a positively skewed distribution, it was first transformed to square root AAO (sqrtAAO) to approach normality. Lifetime number of depressive episodes was a count variable; therefore, we considered Poisson and negative binomial (NB) models as most suitable in subsequent analyses. Episode frequency was defined as the rate of the lifetime number of depressive episodes over the total duration of MDD (=age – age at onset). As its distribution was also highly skewed, a natural logarithm transformation (lnepisfreq) was used for this dependent variable in fitted linear models.

### Investigation of familial effects

To investigate the familiality of AAO in the DeNt siblings sample, we initially fitted a three-level linear mixed model (LMM) with sqrtAAO as the dependent variable, sex as fixed effect covariate and center and family as random effects (subjects nested within families; families nested within centers). Familiality of sqrtAAO was documented if the variance of the random effect of family was significantly greater than zero. The strength of the familial effect was measured by calculating the family-level residual intraclass correlation coefficient (ICC) and its confidence interval (CI) (Wynants *et al.* 2013); the residual ICC represents the proportion of the residual variance of sqrtAAO (after taking into account the effect of fixed covariates – in this case sex) accounted for by family membership. As it has been suggested that AAO can be considered a censored variable and siblings correlated in chronological age will also tend to be correlated in AAO (Schulze *et al.* 2006), we also fitted a model additionally including age as a fixed effects covariate and calculated the significance of the family random effect as well as the ICC. Analyses were implemented with the Stata v. 13 (StataCorp, 2013) *mixed* command.

To investigate the familiality of episodicity in the DeNt siblings sample, we employed two methods. In the first method, we fitted a two-level NB generalized linear mixed model (GLMM) with episode count as the dependent variable, sex, age and center as fixed effects covariates, (ln)MDD duration as offset variable (this reflects the time over which the count response is generated) and family as random effect (subjects nested within families; families nested within centers). Analysis was performed with the Stata v. 13 *menbreg* command, with adaptive Gauss–Hermite quadrature as integration method (seven integrations points) (Bolker *et al.* 2009). Familiality of episodicity was similarly assessed by testing whether the variance of the family random effect was significantly greater than zero. The family-level ICC for episodicity and its CI, i.e. the proportion of the variance of episode frequency explained by family membership after taking into account the effect of center, were finally calculated in a ‘reduced’ version of our model, i.e. one without any subject-level covariates (sex, age), on the basis of recent work formulating an ICC in GLMMs for overdispersed count data (see Supplementary Method) (Carrasco, 2010). We also used a second method to investigate the familiality of episodicity as it was previously used to investigate the familial variation of episode frequency in bipolar disorder (Fisfalen *et al.* 2005); we fitted a three-level LMM with lnepisfreq as the dependent variable, sex and age as fixed effects covariates, and center and family as random effects (subjects nested within families; families nested within centers). Familiality of lnepisfreq was similarly investigated. A family-level residual ICC and its CI were finally calculated.

### Inference of narrow-sense heritability from familiality

Observed phenotypic covariance between relatives (familiality) can be partitioned to causal genetic and environmental covariance components; for full siblings, phenotypic covariance is the sum of half their additive genetic variance plus a quarter of the dominance variance plus the shared environment variance (the contribution of epistatic interactions is ignored) (COV_FS_ = 1/2*V*_A_ + 1/4*V*_D_ + *V*_C_) and their phenotypic correlation is the ratio of their phenotypic covariance by the phenotypic variance (ICC = COV_FS_/*V*_P_) (Falconer & Mackay, 1996; Tenesa & Haley, 2013). Therefore, twice the full-siblings correlation (2 × ICC) is a good estimate of narrow-sense heritability (*V*_A_/*V*_P_) only when dominance and shared environment contributions are assumed negligible and can hence provide an upper bound estimate of heritability (‘maximal heritability’).

### Assessment of SNP heritabilities

To investigate the SNP heritability of AAO in genotyped cases (merged RADIANT and GSK Munich samples), we first fitted a LMM with sqrtAAO as the dependent variable, sex and study as fixed-effects covariates and center as random effect; we then saved the residuals. Analysis was performed with the Stata v. 13 *mixed* command. The model residuals were then used to calculate the SNP heritability of AAO with the GREML method in GCTA software, using 10 principal components as covariates, a genetic relationship matrix (GRM) cut-off of 0.025 and a minor allele frequency (MAF) cut-off of 0.01.

To investigate the SNP heritability of episodicity in genotyped cases, we employed two methods to obtain an adjusted episodicity variable. In the first method, we fitted a NB GLMM (Stata v. 13 me*nbreg* command) with episode count as the dependent variable, sex, age and study as fixed-effects covariates, center as random effect, (ln)MDD duration as offset variable, and saved episode frequency deviance residuals. As their distribution was slightly skewed, we rank-normalized them using Blom's formula (Blom, 1958). The rank-normalized adjusted episodicity residuals were then used to calculate the SNP heritability of episodicity with GCTA software, using the same specifications as above. In the second method, we fitted a LMM with lnepisfreq as the dependent variable, sex, age and study as fixed-effects covariates, and center as random effect. We then saved the residuals, rank-normalized them using Blom's formula, and finally used them with GCTA software as previously described.

We finally estimated the SNP heritabilities of AAO and episodicity that could be detected with a power of 80% (Visscher *et al.* 2014).

## Results

**Fig. 1.**
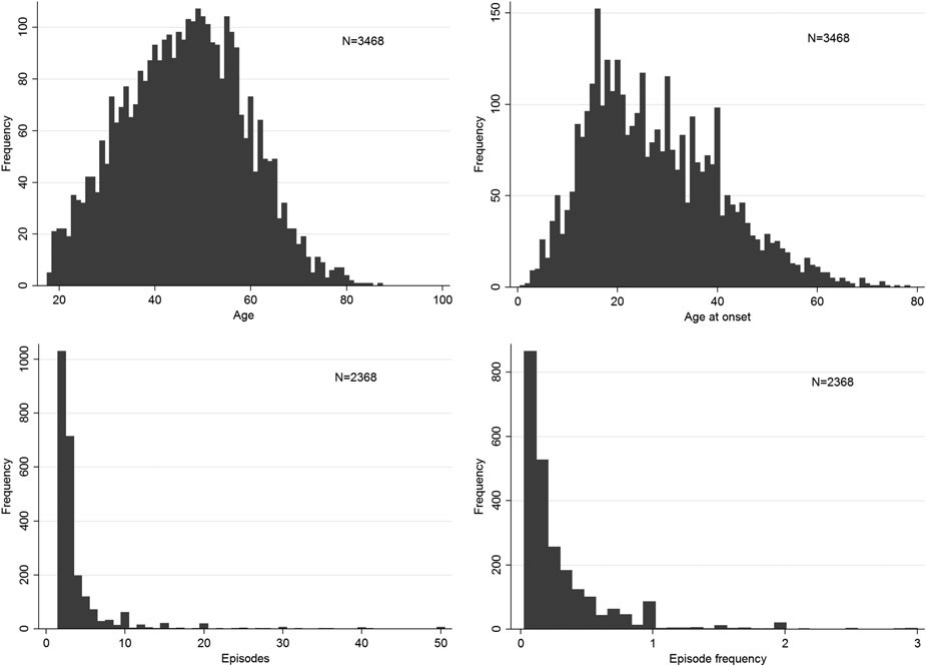
Frequency distributions (histograms) of (*a*) age, (*b*) age at onset, (*c*) episode count and (*d*) episode frequency in genotyped cases (merged RADIANT and GSK Munich samples).

Demographic and clinical characteristics of DeNt siblings and genotyped cases (RADIANT and GSK Munich samples and their merge) are shown in Table 2 and Supplementary Table S1. Frequency distributions (histograms) of age, AAO, episode count and episode frequency in genotyped cases (Fig. 1, Supplementary Fig. S2) and in DeNT siblings (Supplementary Fig. S1) were plotted.

**Table 2.** Demographic and clinical characteristics of DeNT siblings and genotyped cases (merged RADIANT and GSK Munich samples)

	DeNt siblings (*N* = 1498)	Genotyped cases (*N* = 3468)
Sex (females)	73.4% (*N* = 1498)	69.8% (*N* = 3468)
Age (yr)	44.8 ± 11.9 (18–80) (*N* = 1498)	46.2 ± 12.7 (18–87) (*N* = 3468)
Age at onset (yr)	22.8 ± 11.2 (0–74) (*N* = 1403)	27.6 ± 13.2 (1–78) (*N* = 3468)
MDD duration (yr)	22.1 ± 12.9 (0–68) (*N* = 1403)	18.7 ± 13.7 (0–71) (*N* = 3468)
Episode count	5.5 ± 8.2 (2–100) (*N* = 898)	4.0 ± 4.7 (2–50) (*N* = 2368)
Episode frequency (episodes/yr)	0.32 ± 0.36 (0.03–3.0) (*N* = 878)	0.29 ± 0.35 (0.03–3.0) (*N* = 2368)

Quantitative data are presented as mean±s.d. (range).

### Familiality of AAO and episode frequency

In the LMM for sqrtAAO in the DeNt sample (*N* = 1403), the variance of the family random effect was significantly greater than zero; the family-level residual ICC was 0.278 (95% CI 0.185–0.395) (Table 3, Supplementary Table S2a). When we additionally included age as a fixed-effects covariate in our model, we obtained a lower but still significant family-level residual ICC of 0.229 (95% CI 0.140–0.354) (Table 3, Supplementary Table S2b).

**Table 3.** Familiality of age at onset (AAO) and episodicity (two methods) in the DeNt affected full-siblings sample

Dependent variable	Fixed effects	Random effects	Log-likelihood	Likelihood ratio test (one-tailed χ^2^, df = 1)	*p* value	ICC
AAO^a^	Sex	Center, family	−2108.30	29.32	<0.001	0.278^d^
		Center	−2122.96			
	Sex, age	Center, family	−2040.70	16.11	<0.001	0.229^d^
		Center	−2048.75			
Episodicity (NB GLMM)^b^	Sex, age, center	Family	−2310.95	80.47	<0.001	0.074^e^
		None	−2351.18			
Episodicity (LMM)^c^	Sex, age	Center, family	−1043.51	7.56	0.003	0.167^d^
		Center	−1047.29			

a Linear mixed model with sqrtAAO as dependent variable.

b Negative binomial generalized linear mixed model (NB GLMM) with episode count as dependent variable and (ln)MDD duration as offset variable.

c Linear mixed model (LMM) with ln(episode frequency) as dependent variable.

d Residual intraclass correlation coefficient (ICC) calculated in the ‘full’ model with all covariates.

e ICC calculated in a model without subject-level covariates (‘reduced’ model, i.e. without sex and age) (Carrasco, 2010).

Our NB GLMM for episodicity in the DeNt sample (*N* = 878) fitted the observed data better than a corresponding Poisson model (Supplementary Table S3). The variance of the family random effect was significantly greater than zero in both the ‘full’ and the ‘reduced’ model (Table 3, Supplementary Table S3). The family-level ICC for episodicity and its CI were calculated on our ‘reduced’ model (online Supplementary Table S3b). We recorded the overdispersion (alpha) parameter and the variance of the family random effect; we also calculated center variance and episode frequency marginal expectation over families. The family-level ICC for episodicity was finally estimated with the Stata v. 13 *nlcom* command using all aforementioned parameter estimates on the basis of Carrasco's formulae (full details in Supplementary Method); ICC = 0.074, s.e. = 0.012 (95% CI 0.051–0.096).

In the LMM for lnepisfreq in the DeNt sample, the variance of the family random effect was significantly greater than zero; the family-level residual ICC was 0.167 (95% CI 0.089–0.292) (Table 3, Supplementary Table S4).

### Calculation of ‘maximal heritability’ from familiality (ICC estimates)

Assuming dominance and shared environment variance components are negligible, narrow-sense heritability reaches its upper limit (‘maximal heritability’), which is twice the ICC estimate. Maximal heritability estimates obtained are 0.46 and 0.56 for AAO (depending on whether age was included as covariate in the model or not, respectively) and 0.15 and 0.33 for episodicity (depending on the method of calculation used, NB GLMM or LMM, respectively) (Table 4).

**Table 4.** Maximal heritability and SNP heritability estimates for age at onset (AAO) and episodicity in MDD

	Maximal heritability (s.e.)^a^	SNP heritability (s.e.)^b^
(sqrt)AAO	0.56 (0.11)0.46 (0.11)^c^	0.17 (0.10)–
Episodicity^d^	0.15 (0.02)0.33 (0.10)	0.09 (0.14)0.13 (0.15)

a Maximal heritabilities, calculated as twice the ICC estimates from the DeNt affected full-siblings sample, provide an *upper limit* to narrow-sense heritability.

b Single nucleotide polymorphism (SNP) heritabilities in genotyped cases (merged RADIANT and GSK Munich samples) provide a *lower limit* to narrow-sense heritability.

c Two estimates are provided depending on whether age was included as covariate (lower line) in the model or not (upper line).

d Two estimates are provided depending on the method of calculation used (negative binomial generalized linear mixed model, upper line; linear mixed model, lower line; full details in text).

### SNP heritability of AAO and episode frequency

In the LMM for sqrtAAO (*N* = 3468), we saved the residuals and then used them with the GREML procedure in GCTA software (Supplementary Table S5). The SNP heritability of sqrtAAO was calculated at 0.175 (s.e. = 0.104, *p* = 0.042).

In the NB GLMM of episodicity (*N* = 2368), we saved episode frequency deviance residuals, rank-normalized them and then used them with the GREML procedure in GCTA software (Supplementary Table S6). The SNP heritability of episodicity was estimated at 0.092 (s.e. = 0.143, *p* = 0.260).

In the LMM of lnepisfreq, we saved lnepisfreq residuals, rank-normalized them and then used them with the GCTA software (Supplementary Table S7). The SNP heritability of episodicity was estimated at 0.129 (s.e. = 0.148, *p* = 0.192).

Power was 0.8 to detect a SNP heritability of 0.25 for AAO and 0.37 for episodicity.

## Discussion

Stratifying MDD cases by AAO and recurrence has extensively been used in MDD genetic research as early-onset and recurrent forms have been repeatedly associated with higher familial aggregation and heritability of MDD (Sullivan *et al.* 2000). However, there is paucity of evidence regarding the familiality and heritability of these MDD subphenotypes. By contrast, several other subphenotypes, such as symptom clusters or dimensions, illness chronicity, personality traits, subaffective temperament profile or suicidality, have been shown to be familial or heritable in MDD (Farmer *et al.* 2003; Dikeos *et al.* 2004; Korszun *et al.* 2004; Mondimore *et al.* 2006; McGirr *et al.* 2009; Aguiar Ferreira *et al.* 2013; Lai *et al.* 2013). Research into the genetic correlates of MDD subphenotypes might help elucidate the pathogenesis of MDD itself, given that it is phenotypically and genetically heterogeneous. Furthermore, assuming that specific subphenotypes are familial and heritable, one could further explore their pleiotropic cross-phenotype genetic correlations with other psychiatric disorders, medical co-morbidities, personality traits or environmental stressors (Lee *et al.* 2012; Solovieff *et al.* 2013).

This is the first study to systematically investigate the familiality of AAO and episodicity in MDD. The two subphenotypes were analyzed as continuous traits in mixed models, which allowed us to investigate their familial clustering as well as the strength of intra-familial correlations (ICC). To investigate the familiality of episodicity, we fitted two alternative models; a NB GLMM for episode count and a simpler LMM for ln(episode frequency). To calculate the ICC of episodicity in the first model, we relied on a modification of a novel, recently published statistical formulation of an ICC in GLMMs for overdispersed count data (Carrasco & Jover, 2003, 2005; Carrasco, 2010). Significant familiality of both AAO and episodicity was found in the DeNt affected full siblings sample. A moderate proportion of the variance of (sqrt)AAO (ICC = 0.23 with age adjustment and ICC = 0.28 without age adjustment) was attributed to family membership. Analysing episodicity, both methods produced small ICC estimates (0.07 and 0.17, respectively). However, since the number of depressive episodes is a count variable, we consider the NB GLMM method as more precise while the LMM method produces inflated estimates.

The heritability of AAO and episodicity has, similarly, rarely been studied; negligible estimates have been reported for both subphenotypes in a small female-only twin study (Kendler *et al.* 1992). We calculated an upper bound estimate of narrow-sense heritability (‘maximal heritability’) of AAO and episodicity in MDD from respective ICC estimates (Table 4). AAO seems to be under stronger additive genetic control than episodicity. Almost half of the variance of AAO and the greatest part of the variance of episodicity are, however, controlled by unique environment and stochastic events. Yet, it is highly probable that gene–environment interactions, which are difficult to disentangle from unique environmental effects (Purcell, 2002; Purcell & Sham, 2002), also contribute. Furthermore, part of the additive genetic variance component of our subphenotypes might in fact be contributed by genetic control of environmental exposure (Kendler & Karkowski-Shuman, 1997; Power *et al.* 2013).

We finally calculated the SNP heritability of the two subphenotypes in unrelated MDD subjects with the GREML procedure in GCTA software (Yang *et al.* 2011). This represents a lower limit to narrow-sense heritability by estimating its component which is captured by common variants (Zaitlen & Kraft, 2012). The SNP heritability of AAO was small but significant (0.17, *p* = 0.04). Estimates were non-significant for episodicity (0.09 and 0.13, depending on the calculation method) (Table 4); our study lacked power to detect significant and accurate estimates of this magnitude, and much larger studies will be required (Visscher *et al.* 2014).

Our AAO analysis used AAO as a continuous outcome variable, but time-to-event (survival) analysis could alternatively be used, which would also take into account controls who have not manifested depression at their age at interview; Cox regression models with random effects (also known as ‘shared-frailty models’) have been described (Therneau & Grambsch, 2000). Although such an analysis would be feasible for genotyped subjects after adding controls, few unaffected DeNt siblings were recruited and we, therefore, opted for the analysis of AAO as a quantitative trait in both datasets so that maximal heritability and SNP heritability estimates thence derived could be comparable.

Similarly, survival models for recurrent events (multiple-failure data) could be used to analyze episodicity (Baethge & Schlattmann, 2004). These models allow for the effects of prior episode number, therapeutic interventions, episode duration, episode-dependent covariates and unobserved heterogeneity (frailty) in recurrence-prone tendency on the risk of future recurrences (Kessing *et al.* 1999; Solomon *et al.* 2000) but could not be applied here as our data lacked information on timing and duration of each episode. Our NB model assumes that episodes are independent events while a semi-parametric survival model would withhold any assumptions about the distribution of episode count; the results of our analysis might, therefore, be subject to some bias.

Two genome-wide studies of the genetic architecture of AAO and episodicity in the RADIANT sample have been published. A GWAS of AAO, analyzed with three different methods, produced no genome-wide significant findings; a non-replicated genome-wide association with the *TUSC3* gene in young males with an early AAO was recorded (Power *et al.* 2012). A preliminary estimate of 0.55 (s.e. = 0.27, *p* = 0.02) for the SNP heritability of (sqrt)AAO was also reported in this study. This earlier estimate, which is substantially higher than our current estimate, was based on AAO in UK cases only, and unadjusted for the effects of sex and cohort (study and center); in the present study, we adjusted sqrtAAO for these covariates, and extended the analysis to MDD cases ascertained from sites across Europe (including the UK) and one site in St Louis, USA. Similarly, a GWAS of episodicity in the RADIANT sample produced only non-replicated findings of suggestive significance; interestingly, polygenic profile analyses based on the PGC MDD and bipolar studies showed that, in subjects with positive family history of MDD, episodicity was predicted by both MDD and bipolar polygenes (Ferentinos *et al.* 2014). These preliminary studies suggest that investigations in larger collaborative samples are certainly warranted.

Bipolar disorder, unlike MDD, shows evidence of the familiality and heritability of AAO and episodicity subphenotypes. Available studies are either based on extended families ascertained through bipolar probands and including relatives with major affective disorders or on clusters of bipolar siblings. AAO is familial, with reported ICC estimates of 0.1 (Schulze *et al.* 2006), 0.29 (O'Mahony *et al.* 2002) and 0.42 (Leboyer *et al.* 1998), while affected siblings of early-onset (AAO£21 years) probands were 4.5 times more likely than others to have an early onset (Lin *et al.* 2006). AAO was estimated to have a heritability of 0.52 in 27 extended families ascertained for bipolar disorder (Visscher *et al.* 2001). Episode frequency was significantly correlated among bipolar probands and their affected relatives with major affective disorders (ICC = 0.56) (Fisfalen *et al*. 2005), refuting a previously reported much lower estimate of 0.15 which did not survive multiple testing correction (O'Mahony *et al.* 2002). Other subphenotypes have been shown to be familial in bipolar disorder, including psychotic symptoms, sleep disturbances, proportion of manic to depressive episodes, polarity at illness onset, suicidality, comorbidities (panic disorder, alcohol and substance abuse), and the quality of social relations (MacKinnon *et al.* 1997; Potash *et al.* 2001; O'Mahony *et al.* 2002; Dikeos *et al.* 2004; Kassem *et al.* 2006; Schulze *et al.* 2006; Lai *et al.* 2013).

Our study comes with some additional limitations. First, episode count was retrospectively self-reported during the SCAN interview without validation from external sources; it may be subject to recall bias and be influenced by the patient's current mood state or personality characteristics (neuroticism, histrionic traits). Second, while maximal heritabilities of AAO and episodicity were derived from phenotypic correlations in a sample with familial MDD (DeNt siblings), SNP heritabilities were assessed in the merged RADIANT and GSK Munich sample where 54.4% of subjects had positive family history of MDD in first-degree relatives; low power did not allow us to recalculate SNP heritabilities for this specific subset. Third, ICC estimates were calculated in DeNt siblings suffering from recurrent MDD, and the SNP heritability of episodicity was assessed in 2368 genotyped cases with recurrent MDD; on the other hand, the SNP heritability of AAO was assessed in 3468 cases, of which 92% had recurrent MDD. Therefore, since heritabilities of AAO and episodicity might be different in familial *v.* non-familial MDD and in recurrent *v.* single episode forms, Table 4 should be interpreted with caution.

In conclusion, this study systematically investigated the familiality of AAO and episodicity in a sample of full siblings with recurrent MDD. Significant familiality of both was found; the strength of the familial effect was moderate for AAO and low for episodicity. An estimate of the upper limit to the narrow-sense heritability of the two subphenotypes was calculated from ICC values. AAO is under stronger additive genetic control than episodicity. We also estimated in unrelated MDD subjects the proportion of the variance of AAO explained by common SNPs (SNP heritability) with the GREML procedure in GCTA software. Analysis was underpowered for calculating SNP heritability of episodicity, confirming the need for larger samples. The statistical framework described here could be useful in future analyses. Assuming there is a substantial genetic basis for AAO and episodicity, one could further explore their pleiotropic genetic correlations with various other traits or conditions in order to unravel additional aspects in the pathogenesis, onset and course of MDD.

## Supplementary material

For supplementary material accompanying this paper visit http://dx.doi.org/10.1017/S0033291715000215.
